# Characterization of the complete mitochondrial genome of the common smoothhound shark, *Mustelus mustelus* (Carcharhiniformes: Triakidae)

**DOI:** 10.1080/23802359.2018.1507642

**Published:** 2018-08-28

**Authors:** Kelvin L. Hull, Simo N. Maduna, Aletta E. Bester-van der Merwe

**Affiliations:** Molecular Breeding and Biodiversity Group, Department of Genetics, Stellenbosch University, Stellenbosch, Western Cape, South Africa

**Keywords:** Carcharhiniformes, smoothhound, mitogenome, Mustelus mustelus, phylogenetic analysis

## Abstract

We present the complete mitochondrial genome of the common smoothhound , *Mustelus mustelus,*which is 16,755 bp long, contains 13 protein-coding genes, 22 tRNA genes, 2 rRNA genes, and non-coding control region. All protein-coding genes begin with the ATG codon, except for the *COI* gene, which begins with GTG. Six protein-coding genes terminated with the TAA codon, and six with incomplete codons, T or TA. The phylogenetic reconstruction places *M*. *mustelus* within the genus *Mustelus*, with the closest relationship to the placental species, *M*. *griseus*. This mitogenome provides valuable information to further unravel the evolution of alternate reproductive modes within the genus.

The common smoothhound, *Mustelus mustelus* (Linnaeus 1758), is a medium-sized epibenthic shark, distributed from the Mediterranean and eastern Atlantic to the south-west Indian Ocean (Weigmann 2016), with the reproduction mode being placental viviparity (Saidi et al. 2008).

In this study, a specimen was collected by the Department of Agriculture, Forestry and Fisheries, South Africa during tagging surveys off the south-west coast of South Africa (Langebaan Lagoon, Western Cape, latitude: 33°06′ S, longitude: 18°01′ E). Total genomic DNA was extracted (Elasmobranch Genetics, Lab 242, SU) from a fin clip sample (LL5MM) using a cetyltrimethylammonium bromide extraction protocol of Sambrook and Russell (2001) and sent to the Agricultural Research Council Biotechnology Platform, South Africa for high throughput sequencing. One microgram of genomic DNA was used for 2 × 250 bp paired-end library preparation with a mean insert size of 400 bp using the Illumina TruSeq^®^ DNA library preparation kit (Illumina). The library was sequenced on two lanes of an Illumina HiSeq™ 2000 sequencer.

The generated reads were submitted to quality control as per Maduna et al. (2017). Mitochondrial sequences were filtered from the data set by conducting a BLASTn search (maximum E-value 1.0 E − 20) against the mitogenome of *M*. *griseus* (Genbank accession NC_023527.1, Chen et al. 2016) in Geneious v10.1 (Kearse et al. 2012). These putative mitochondrial sequences were mapped against the mitogenome of *M*. *griseus* using the Geneious Read Mapper algorithm, with default parameters. The mitogenome was then annotated using the MitoFish and MitoAnnotator web service (Iwasaki et al. 2013).

To infer the phylogenetic placement of *M*. *mustelus*, the mitogenome of the species (Genbank Accession MH559351) was aligned against 31 complete mitogenomes representing the eight orders of sharks (Figure 1) using MAFFT (Katoh and Standley 2013), with default parameters. The nucleotide substitution model that best fit the alignment was determined in JMODELTEST v2.0 (Darriba et al. 2012) according to the Bayesian Information Criterion, with the GTR + I+G model being the best fit. Bayesian inference of the phylogenetic relationships among mitogenomes was performed in MRBAYES v3.2.6 (Ronquist et al. 2012) with 2,000,000 MCMC generations and the first 500,000 generations discarded as burn-in, performed through the CIPRES Science Gateway (Miller et al. 2010). The consensus tree (Figure 1) was visualized in FIGTREE v1.4.3 (http://tree.bio.ed.ac.uk/software/figtree).

**Figure 1.**
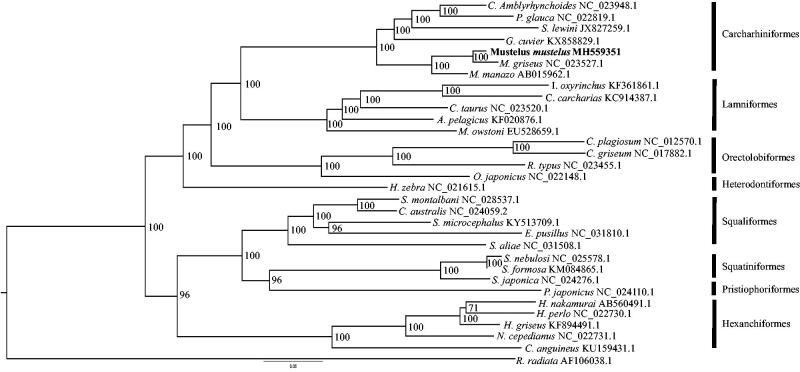
Phylogenetic relationships between the eight orders of shark constructed using Bayesian inference from 31 whole mitogenome sequences, with the thorny skate (*Raja radiata*) mitogenome included as an outgroup. Numbers at nodes indicate posterior probabilities. Genbank accession numbers are given adjacent to the species name, and scale bar indicates groupings of species into orders.

The assembled mitogenome of *M*. *mustelus* is 16,755 bases in length and displayed synteny with other shark and broader vertebrate mitogenomes. The overall base composition of the genome was: A: 31%, T: 30%, C: 25%, and G: 14%. The phylogenetic reconstruction shows the phylogenetic placement of *M*. *mustelus* within the genus *Mustelus* with the closest relationship to the placental species, *M*. *griseus*. The positioning of the species could be explained by reproductive mode, as *M*. *mustelus* and *M*. *griseus* are viviparous placental, in contrast to *M*. *manazo*, which is aplacental (Teshima and Koga 1973; Smale and Compagno 1997). Therefore, the mitogenome for *M*. *mustelus* is an important resource for future conservation and evolutionary biology research such as in-depth phylogenetic placement of species of *Mustelus* with alternate modes of reproduction.
